# Subtype-specific analysis of factors associated with assisted reproductive technology indication and live birth in patients with adenomyosis: a retrospective study

**DOI:** 10.1007/s00404-026-08490-4

**Published:** 2026-06-26

**Authors:** Kota Tanaka, Yosuke Ono, Hajime Ota, Dai Miyashita, Tatsuya Yoshihara, So Owada, Satoko Sasatsu, Maki Ogi, Yasuhiko Okuda, Hideto Yamada, Shinichiro Wada, Osamu Yoshino

**Affiliations:** 1 Department of Obstetrics and Gynecology, University of Yamanashi, 1110 Shimokatou, Chuo City, Yamanashi Prefecture 409-3898 Japan; 2 Department of Obstetrics and Gynecology, Teine Keijinkai Hospital, 1-40, 12-chome, Maeda, Teine-ku, Sapporo-shi, 006-8555 Japan; 3 Center for Recurrent Pregnancy Loss, Teine Keijinkai Hospital, 1-40, 12-chome, Maeda, Teine-ku, Sapporo-shi, 006-8555 Japan

**Keywords:** Adenomyosis, Endometriosis, Live birth, Magnetic resonance imaging, Pregnancy, Subtype

## Abstract

**Purpose:**

The magnetic resonance imaging (MRI)-based classification of adenomyosis subtypes helps predict reproductive and obstetric outcomes; however, background factors associated with use of assisted reproductive technology (ART) and live birth within each individual subtype remain unclear.

**Methods:**

We conducted a multicenter retrospective study of 199 premenopausal women (32–49 years) who underwent pelvic MRI and laparoscopic surgery and had histopathologic confirmation of adenomyosis (January 2010–May 2023). Patients were classified as intrinsic (n = 58), extrinsic (n = 61), or indeterminate (n = 80) subtype. Within each subtype, multivariate logistic regression tested independent associations of age, ART history, gravidity, parity, lesion thickness, and intraoperative findings—including pelvic endometriosis—with ART use and live birth.

**Results:**

The extrinsic subtype had a higher proportion with ART history than the intrinsic subtype (32.8% vs 8.6%; p = 0.008). Live birth rate was lower in the indeterminate than the intrinsic subtype (60.0% vs 86.2%; p = 0.0048). In the extrinsic subtype, greater lesion thickness independently predicted lower odds of live birth (adjusted OR = 0.94 per 1-mm increase; 95% CI, 0.88–0.99; p = 0.048). In the indeterminate subtype, older age was associated with ART use (adjusted OR = 1.155 per year; 95% CI, 1.002–1.351; p = 0.047), and ovarian endometrioma was linked to reduced live birth (adjusted OR = 0.172; 95% CI, 0.054–0.508; p = 0.001). In the intrinsic subtype, women with live birth were older than those without, but age was not an independent factor.

**Conclusion:**

Adenomyosis lesion thickness and coexisting endometriosis are associated with ART indication and live birth outcomes in a subtype-specific manner and may support individualized counselling and management.

**Supplementary Information:**

The online version contains supplementary material available at https://doi.org/10.1007/s00404-026-08490-4.

## What does this study add to the clinical work?

**Table:** 

MRI-defined adenomyosis subtypes show distinct factors associated with ART indication and live birth. Subtype-specific assessment of lesion burden and coexisting endometriosis may improve fertility counselling and individualized reproductive management.	

## Introduction

Adenomyosis is a benign gynecological condition characterized by the presence of ectopic endometrial glands and stroma within the myometrium [1]. Commonly manifesting with symptoms such as dysmenorrhea, menorrhagia, and chronic pelvic pain, this condition causes significant quality of life (QOL) reduction in women of reproductive age [2]. In recent years, accumulating evidence has demonstrated that adenomyosis not only contributes to infertility [3–5] but is also associated with increased obstetric complication risks—specifically fetal growth restriction, placenta previa, and postpartum hemorrhage [6–8]. Although hysterectomy remains the definitive treatment for symptom resolution, it is unsuitable for women who desire to preserve fertility. Consequently, there is a growing clinical need to establish effective fertility-preserving strategies. However, there is currently no consensus on the optimal therapeutic approach, largely due to the heterogeneity of the disease in terms of its extent, location, and underlying pathophysiology [9, 10].

Recent advances in non-invasive imaging, particularly magnetic resonance imaging (MRI), have enabled more precise evaluation of adenomyosis. This imaging method can assess not only the presence of lesions but also their location, extent, and morphological features [11]. This has led to the emergence of MRI-based classification systems, which stratify adenomyosis into subtypes—such as intrinsic, extrinsic, and indeterminate—based on the relationship between lesions and the endometrial–myometrial junction [12, 13]. Recent studies suggest that these subtypes may differ significantly in their association with clinical outcomes, including infertility treatment response and live birth rates, indicating potential for individualized treatment planning [14, 15]. However, it remains unclear whether MRI-defined adenomyosis subtypes represent distinct disease entities or simply reflect different manifestations observed during the progression of the same disease.

Although MRI has been reported to have higher diagnostic specificity than ultrasound, its diagnostic accuracy remains limited [15]. Adenomyosis frequently coexists with pelvic endometriosis—particularly deep endometriosis (DE)—which MRI may not detect with high sensitivity. Furthermore, MRI cannot assess pelvic adhesions or intraoperative anatomical distortions. Therefore, a comprehensive evaluation that combines MRI, histopathological confirmation, and intraoperative findings is essential for the accurate delineation of subtype-specific clinical implications. To date, not many studies have examined fertility outcomes across MRI-based subtypes of adenomyosis with detailed surgical and pathological validation. Furthermore, little is known about the specific clinical factors that may be associated with assisted reproductive technology (ART) utilization and live birth outcomes within each subtype.

In this retrospective study, we aimed to bridge this knowledge gap by analysing a cohort of women with pathologically confirmed adenomyosis who underwent both preoperative MRI and laparoscopic surgery. The primary objective of the study was to identify subtype-specific factors that are independently associated with ART implementation and the likelihood of achieving a live birth. By focusing on individualized features within each MRI-defined subtype, this study seeks to support more precise, tailored approaches to fertility preservation in women affected by adenomyosis.

## Materials and methods

This retrospective study was approved by the institutional review boards of the University of Yamanashi Hospital and Teine Keijinkai Hospital, and the study was conducted in accordance with the principles of the Declaration of Helsinki (ethics approval No.: CS 0013). Due to the retrospective nature of the study, the requirement for written informed consent was waived. This study was designed as a retrospective, cross-sectional analysis; therefore, the present study does not assume a longitudinal progression among adenomyosis subtypes but instead focuses on cross-sectional differences in reproductive characteristics assessed at the time of MRI-based diagnosis.

We initially enrolled 230 premenopausal women aged < 50 years who underwent surgery for clinically diagnosed adenomyosis—either hysterectomy or adenomyomectomy—between January 2010 and May 2023 at the two participating institutions. All cases were histopathologically confirmed as adenomyosis following surgery. Typical MRI features used to diagnose adenomyosis included thickening of the junctional zone (generally ≥ 12 mm), ill-defined areas of low signal intensity within the myometrium on T2-weighted images, and the presence of small hyperintense myometrial foci representing ectopic endometrial glands or microcysts. Additional supportive findings included asymmetrical myometrial thickening and punctate high-signal areas on T1-weighted images suggestive of hemorrhagic components. MRI has been widely accepted as a reliable noninvasive modality for the diagnosis of adenomyosis, and these imaging characteristics are considered representative of the disease and have been extensively described in radiologic–pathologic correlation studies [16, 17]. Hormonal treatments, such as GnRH agonists, GnRH antagonists, or progestins, were administered in many patients; however, all of these treatments were initiated after the preoperative MRI examinations, implying that they did not influence MRI-based subtype classification.

Among the initially enrolled patients, a subset did not demonstrate these characteristic MRI findings on preoperative imaging. Because the absence of typical MRI features precluded reliable imaging-based diagnosis and accurate subtype classification, these cases were excluded from the final analysis. Accordingly, the final study population comprised 199 women who had both histopathological confirmation of adenomyosis and preoperative MRI findings sufficient to support imaging diagnosis and subtype classification based on previously reported criteria.

MRI-based classification of adenomyosis subtypes was performed according to previous reports [12], and lesions were categorized into three subtypes: (a) the intrinsic type, located adjacent to the endometrial side of the myometrium; (b) the extrinsic type, located toward the serosal side of the uterus; and (c) the indeterminate type, defined as diffuse or transmural lesions that could not be clearly classified as intrinsic or extrinsic. MRI images were independently reviewed by two board-certified obstetricians and gynecologists, and only cases with concordant subtype classification were included in the analysis. A small number of cases (n = 17) exhibited adenomyotic lesions localized within the myometrium in a fibroid-like pattern, corresponding to subtype III as described by Kishi et al. Because of the limited number of such cases and previous reports suggesting that this subtype has minimal impact on reproductive and obstetric outcomes [12], these cases were excluded from the present analysis.

In addition, patients were excluded if they had incomplete clinical data or relevant comorbidities (including chronic hypertension, diabetes mellitus, endocrine disorders, cardiovascular disease, or malignancy), or if their MRI findings did not allow classification into any adenomyosis subtype. To investigate how adenomyotic lesion burden may influence ART use and pregnancy outcomes, the maximum lesion thickness in both the anterior and posterior uterine walls was measured on sagittal MRI slices, and their sum was used as a surrogate index of lesion volume. Ovarian endometriomas were also evaluated on MRI. A diagnosis was made when a lesion showed high signal intensity on T1-weighted images and low signal intensity on T2-weighted images (shading sign), with a maximum diameter of ≥ 3 cm.

This study aimed to identify subtype-specific factors associated with ART usage and live birth outcomes. The following clinical data were retrospectively collected from medical records: age, body mass index (BMI), gravidity, parity, history of recurrent pregnancy loss (defined as ≥ 2 miscarriages or stillbirths, excluding elective abortions), indications for ART (defined as failure to achieve pregnancy with conventional infertility treatments; cases in which ART was indicated solely due to male factor infertility were excluded), history of ART, presence of ovarian endometrioma or pelvic endometriosis confirmed intraoperatively, presence of uterine fibroids, and symptoms such as dysmenorrhea and hypermenorrhea.

## Statistical analysis

Statistical analyses were performed using GraphPad Prism version 10.4.2 (GraphPad Software, San Diego, CA, USA). The normality of continuous data distribution was assessed using the Shapiro–Wilk test. For comparisons between two groups, Student’s t-test or the Mann–Whitney U test was used for normally and non-normally distributed continuous variables, respectively. Categorical variables were compared using the chi-square test or Fisher’s exact test, as appropriate. For comparisons among the three adenomyosis subtypes, the one-way analysis of variance (for normally distributed data) or the Kruskal–Wallis test (for non-normally distributed data) was used for continuous variables, and the chi-square test or Fisher’s exact test was used for categorical variables. When significant differences were detected among the three groups, post hoc pairwise comparisons were performed using Dunn’s multiple comparisons test or Fisher’s exact test with the Bonferroni correction.

Multivariate logistic regression analyses were performed to identify independent predictors of ART usage and live birth. Continuous variables, including age, body mass index (BMI), and adenomyosis lesion thickness, were analyzed as continuous predictors. Categorical variables (e.g., the presence or absence of ovarian endometrioma, uterine fibroids, deep endometriosis, history of ART, recurrent pregnancy loss, and parity) were treated as binary variables. Because endometriosis is a well-recognized confounder that can independently affect implantation and fertility, the presence of ovarian endometrioma and pelvic endometriosis was prespecified as clinically relevant and included as covariates in all multivariate models to account for their potential confounding effects on ART indication and live birth outcomes. Odds ratios (ORs) with 95% confidence intervals (CI) were calculated. ORs for continuous variables are presented per one-unit increment, and ORs for binary variables are presented relative to the reference category.

## Results

### Patient characteristics and comparison among adenomyosis subtypes

The clinical characteristics of the 199 patients classified into MRI-defined adenomyosis subtypes are summarized in Table 1. Of these patients, 58 (29.1%) had the intrinsic subtype, 61 (30.7%) had the extrinsic subtype, and 80 (40.2%) had the indeterminate subtype. There were no significant differences among the three subtypes with respect to age at MRI examination, body mass index (BMI), or the interval between the last pregnancy and MRI. In contrast, several reproductive and disease-related characteristics differed significantly by subtype. Specifically, the proportions of patients with a history of ART use, recurrent pregnancy loss (RPL), prior live birth, ovarian endometrioma, pelvic endometriosis, and adenomyosis lesion thickness showed significant variation across subtypes (all p < 0.05).

**Table 1:** Comparison of clinical characteristics across adenomyosis subtypes classified by MRI findings (intrinsic, extrinsic, and indeterminate subtypes)

	a: Intrinsic (n = 58)	b: Extrinsic (n = 61)	c: Indeterminate (n = 80)	Overall p value	Pairwise comparison (Bonferroni correction): p value
Age	42 (34–48)	41 (34–48)	41 (32–49)	0.174	–
BMI	21.6 (17.9–34.0)	22.6 (19.2–33.9)	22.3 (17.4–35.7)	0.624	–
Gravidity	2 (0–4)	2 (0–5)	2 (0–9)	0.057	–
History of ART	5 (8.6%)	20 (32.8%)	21 (26.3%)	0.0109	a vs b: 0.008, a vs c: 0.050, b vs c: 1.000
RPL	2 (3.4%)	7 (11.5%)	15 (18.8%)	0.0483	a vs b: 0.57, a vs c: 0.045, b vs c: 1.000
History of live-birth	50 (86.2%)	48 (78.7%)	48 (60%)	0.003	a vs b: 1.000, a vs c: 0.0048, b vs c: 0.054
Endometrioma	5 (8.6%)	34 (55.7%)	31 (38.8%)	< 0.001	a vs b: < 0.001, a vs c: < 0.001, b vs c: 0.13
Pelvic endometriosis	11 (19.0%)	38 (62.3%)	26 (32.5%)	< 0.001	a vs b: < 0.001, a vs c: 0.229, b vs c: < 0.001
Myoma	12 (20.7%)	13 (21.3%)	14 (17.5%)	0.827	–
Thickness of adenomyosis (mm)	45 (21.8–85.0)	35 (21.2–93.4)	48.6 (28.8–126.4)	< 0.001	a vs b: 0.020, a vs c: 0.399, b vs c: < 0.001
The interval from the last pregnancy to MRI examination	8 (1–14)	6.8 (1–14)	6 (1–15)	0.414	–
Total laparoscopic hysterectomy	54	49	69	0.209	–
Adenomyomectomy	4	12	11	0.209	–
Dysmenorrhea	51 (87.9%)	55 (90.2%)	75 (93.8%)	0.0285	a vs b: 1.000, a vs c: 1.000, b vs c: 1.000
Hypermenorrhea	49 (84.5%)	47 (77.0%)	74 (92.5%)	0.0649	–

Values are expressed as median (range) for continuous variables and as number (percentage) for categorical variables

Comparisons among the three subtypes were performed using the Kruskal–Wallis test for continuous variables and either the chi-square test or Fisher’s exact test for categorical variables, as appropriate

When overall p-values indicated a significant difference among the groups, pairwise comparisons were conducted using Dunn’s multiple comparisons test for continuous variables, or Fisher’s exact test with Bonferroni correction for categorical variables

Fisher’s exact test was preferentially used for categorical comparisons involving small sample sizes. A p-value < 0.05 was considered statistically significant

When comparing the intrinsic and extrinsic subtypes, patients with the extrinsic subtype showed a markedly higher prevalence of ART history (32.8% vs. 8.6%, p = 0.008), as well as a substantially higher coexistence of ovarian endometrioma (55.7% vs. 8.6%, p < 0.001) and pelvic endometriosis (62.3% vs. 19.0%, p < 0.001). These findings indicate that the extrinsic subtype is more frequently associated with endometriosis-related infertility and a greater need for ART. In contrast, the intrinsic subtype was characterized by a higher proportion of patients with a prior live birth compared with the indeterminate subtype (86.2% vs. 60.0%, p = 0.0048), suggesting relatively preserved reproductive potential in this group. With regard to adenomyosis lesion burden, lesion thickness was greatest in the indeterminate subtype and was significantly larger than that observed in the extrinsic subtype (48.6 mm vs. 35.0 mm, p < 0.001). The intrinsic subtype showed greater lesion thickness than the extrinsic subtype (p = 0.020), but did not differ significantly from the indeterminate subtype.

### Factors associated with ART utilization and live birth in the intrinsic subtype

Among patients with the intrinsic subtype, there were no significant differences in baseline clinical characteristics—including age, gravidity, parity, adenomyosis lesion thickness, or the presence of ovarian endometrioma or pelvic endometriosis—between those with and without a history of ART treatment (Table 2a). When patients were stratified by live birth history, those who achieved at least one live birth were significantly older than those who did not (median 42.5 [34–49] vs. 40.5 [35–46] years, p = 0.02, Table 2b). However, in multivariate logistic regression analysis adjusting for age and other potential confounders, age was not independently associated with live birth (adjusted OR 0.82, 95% CI 0.62–1.07, p = 0.145, Supplementary Table 1).

**Table 2:** Comparison of clinical characteristics according to ART history and live-birth history in patients with intrinsic subtype of adenomyosis

a	Intrinsic subtype	History of ART ( +), n = 5	History of ART (−), n = 53	p value
	Age	42 (35–43)	42 (34–49)	0.391
	BMI	22.6 (18.9–26.0)	21.6 (17.9–30.4)	0.929
	Endometrioma	0 (0%)	5 (9.4%)	1
	Pelvic endometriosis	1 (20.0%)	10 (18.9%)	1
	Myoma	0	12	0.537
	Thickness of adenomyosis (mm)	52 (33–66)	45 (22.8-85)	0.42

b	Intrinsic subtype	History of Live-birth ( +), n = 50	History of Live-birth (−), n = 8	p value
	Age	42.5 (34–49)	40.5 (35–46)	0.02
	BMI	21.7 (17.9–34.0)	21.1 (17.9–27.0)	0.286
	Endometrioma	5 (10%)	0 (0%)	1
	Pelvic endometriosis	9 (18.0%)	2 (25%)	0.639
	Myoma	9 (18.0%)	3 (37.5%)	0.342
	RPL	2 (4.0%)	0 (0%)	0.259
	Thickness of adenomyosis (mm)	46.1 (22.8–85)	39 (21.8–66)	0.212

Continuous variables are shown as median (range) and categorical variables as number (percentage)

Comparisons were made using the Mann–Whitney U test for continuous variables and Fisher’s exact test for categorical variables

A p-value < 0.05 was considered statistically significant

### Factors associated with ART utilization and live birth in the extrinsic subtype

In the extrinsic subtype, patients with a history of ART treatment exhibited significantly greater adenomyosis lesion thickness than those without ART history (median 42.7 [23.5–99.7] mm vs. 31.9 [21.2–71.0] mm, p = 0.037, Table 3a). The prevalence of ovarian endometrioma also tended to be higher in the ART group, although this difference did not reach statistical significance (46.3% vs. 20.0%, p = 0.066). To further clarify independent factors associated with ART utilization in the extrinsic subtype, a multivariate logistic regression analysis was performed. After adjustment for age, body mass index (BMI), the presence of ovarian endometrioma, pelvic endometriosis, uterine fibroids, and adenomyosis lesion thickness, none of these variables were identified as independent predictors of ART utilization. Although ovarian endometrioma showed a tendency toward association with ART use (adjusted OR 3.35, 95% CI 0.85–15.21, p = 0.08, Table 4a), this did not reach statistical significance, suggesting that ART utilization in the extrinsic subtype reflects a multifactorial clinical decision rather than a single dominant determinant. When patients were stratified by live birth history, adenomyosis lesion thickness was significantly greater in patients who did not achieve live birth compared with those who did (42.7 [23.5–99.7] mm vs. 33.4 [21.2–70.9] mm, p = 0.049, Table 3b). Age tended to be lower in the live birth group, although this difference was not statistically significant (41[34–48] vs 43[35–48], p = 0.099, Table 3b). In multivariate analysis, no variables were independently associated with ART utilization. Ovarian endometrioma showed a nonsignificant trend toward increased ART use (adjusted OR 3.35, p = 0.084, Table 4a), whereas adenomyosis lesion thickness was not associated with ART utilization. In contrast, adenomyosis lesion thickness was independently associated with live birth, with each 1 mm increase corresponding to an approximately 6% reduction in the odds of achieving a live birth (adjusted OR = 0.94, 95% CI 0.880–0.990, p = 0.048, Table 4b).

**Table 3:** Comparison of clinical characteristics according to ART history and live-birth history in patients with extrinsic subtype of adenomyosis

a	Extrinsic subtype	ART ( +), n = 20	ART (−), n = 41	p value
	Age	41 (36–47)	41 (34–48)	0.623
	BMI	24.1 (19.4–33.9)	22.0 (18.0–32.5)	0.463
	Endometrioma	15 (75.0%)	19 (46.3%)	0.066
	Pelvic endometriosis	15 (75.0%)	23 (56.1%)	0.251
	Myoma	4 (25.0%)	8 (19.5%)	0.766
	Thickness of adenomyosis (mm)	42.7 (23.5–99.7)	31.9 (21.2–71.0)	0.037

b	Extrinsic subtype	History of live-birth ( +), n = 48	History of live-birth (−), n = 13	p value
	Age	41 (34–48)	43 (35–48)	0.099
	BMI	22.1 (18.0–33.9)	23.7 (19.4–29.7)	0.593
	Endometrioma	25 (52.1%)	9 (69.2%)	0.43
	Pelvic endometriosis	28 (58.3%)	10 (76.9%)	0.336
	Myoma	8 (16.7%)	5 (38.5%)	0.187
	RPL	4 (8.3%)	3 (23.1%)	0.323
	Thickness of adenomyosis (mm)	33.8 (21.2–93.4)	55 (23.5–99.7)	0.049

Continuous variables are shown as median (range) and categorical variables as number (percentage)

Comparisons were made using the Mann–Whitney U test for continuous variables and Fisher’s exact test for categorical variables

A p-value < 0.05 was considered statistically significant

**Table 4:** Multivariate analysis of assisted reproductive technology and live birth in the extrinsic subtype

	Variables	Adjusted-odds ratio (95% confidence interval)	p value
a	Extrinsic subtype; ART		
	Age	1.025 (0.832–1.262)	0.809
	BMI	0.921 (0.771–1.089)	0.336
	Endometrioma	3.352 (0.853–15.206)	0.084
	Pelvic endometriosis	2.004 (0.481–9.206)	0.341
	Myoma	0.743 (0.114–4.102)	0.736
	Thickness of adenomyosis (mm)	0.974 (0.939–1.006)	0.109
b	Extrinsic subtype; Live-birth		
	Age	1.076 (0.843–1.455)	0.571
	BMI	0.955 (0.747–1.184)	0.681
	Endometrioma	0.535 (0.089–2.666)	0.45
	Pelvic endometriosis	0.811 (0.128–4.510)	0.811
	Myoma	0.605 (0.098–4.611)	0.604
	Thickness of adenomyosis (mm)	0.940 (0.880–0.990)	0.048

Odds ratios are presented per 1-unit increase for continuous variables (age, BMI, and lesion thickness of adenomyosis), and relative to the reference category for binary variables (presence or absence of ovarian endometrioma, uterine fibroids, and deep infiltrating endometriosis)

### Factors associated with ART utilization and live birth in the indeterminate subtype

In the indeterminate subtype, patients who underwent ART were significantly younger than those who did not (38 [32–47] vs. 41 [34–49] years, p = 0.026, Table 5a). Pelvic endometriosis was also significantly more prevalent in the ART group (52.4% vs. 25.4%, p = 0.031, Table 5a), whereas adenomyosis lesion thickness did not differ between groups. Regarding live birth outcomes, patients without a history of live birth had a significantly higher prevalence of ovarian endometrioma (62.5% vs. 22.9%, p < 0.001) and pelvic endometriosis (46.9% vs. 22.9%, p = 0.025, Table 5b). In multivariate analysis, older age was independently associated with ART utilization (adjusted OR = 1.16, 95% CI 1.002–1.351, p = 0.047, Table 6a), while the presence of ovarian endometrioma was independently associated with reduced odds of live birth (adjusted OR = 0.172, 95% CI 0.054–0.508, p = 0.001; Table 6b).

**Table 5:** Comparison of clinical characteristics according to ART history and live-birth history in patients with indeterminate subtype of adenomyosis

a	Indeterminate subtype	History of ART ( +), n = 21	History of ART (−), n = 59	p value
	Age	38 (32–47)	41 (34–49)	0.026
	BMI	22.0 (15.8–43.8)	22.5 (17.6–31.6)	0.956
	Endometrioma	12 (57.1%)	19 (32.2%)	0.079
	Pelvic endometriosis	11 (52.4%)	15 (25.4%)	0.031
	Myoma	5 (23.8%)	9 (15.3%)	0.581
	Thickness of adenomyosis (mm)	48.4 (28.8–126.4)	48.8 (29.4–98.8)	0.707

b	Indeterminate subtype	History of live-birth ( +), n = 48	History of live-birth (−), n = 32	p value
	Age	41 (33–49)	40 (32–49)	0.55
	BMI	22.1 (17.6–35.9)	22.9 (15.8–43.8)	0.42
	Endometrioma	11 (22.9%)	20 (62.5%)	< 0.001
	Pelvic endometriosis	11 (22.9%)	15 (46.9%)	0.025
	Myoma	8 (16.7%)	6 (18.8%)	0.952
	RPL	10 (20.8%)	5 (15.6%)	0.77
	Thickness of adenomyosis (mm)	49.1 (28.8– 95.9)	47.5 (29.4–126.4)	0.656

Continuous variables are shown as median (range) and categorical variables as number (percentage)

Comparisons were made using the Mann–Whitney U test for continuous variables and Fisher’s exact test for categorical variables

A p-value < 0.05 was considered statistically significant

**Table 6:** Multivariate analysis of assisted reproductive technology and live birth in the indeterminate subtype

	Variables	Adjusted-odds ratio (95% confidence interval)	p value
a	Indeterminate subtype; ART		
	Age	1.155 (1.002–1.351)	0.047
	BMI	0.981 (0.877–1.106)	0.742
	Endometrioma	1.870 (0.574–6.102)	0.295
	Pelvic endometriosis	2.257 (0.656–7.858)	0.194
	Myoma	2.245 (0.567–8.564)	0.241
b	Indeterminate subtype; Live-birth		
	Age	1.023 (0.893–1.179)	0.743
	BMI	1.078 (0.965–1.217)	0.186
	Endometrioma	0.172 (0.054–0.508)	0.001
	Pelvic endometriosis	0.505 (0.159–1.624)	0.247
	Myoma	0.778 (0.198–3.150)	0.719

Odds ratios are presented per 1-unit increase for continuous variables (age, BMI), and relative to the reference category for binary variables (presence or absence of ovarian endometrioma, uterine fibroids, and deep infiltrating endometriosis)

## Discussion

To the best of our knowledge, this study is the first to classify adenomyosis based on MRI findings into subtypes and to analyze the factors associated with fertility treatments and live birth rates within each subtype. In particular, the extrinsic subtype was frequently associated with ovarian endometriomas and pelvic endometriosis, consistent with previous reports suggesting a high rate of comorbidity with endometriosis [12]. Furthermore, the proportion of patients who had undergone ART was significantly higher for the extrinsic subtype compared to other subtypes, likely reflecting the increased infertility risk associated with endometriosis.

Previous studies have reported an association between adenomyosis and impaired reproductive outcomes, including reduced implantation and live birth rates, particularly in ART cycles [18, 19]. However, reported effect sizes have varied widely, likely due to heterogeneity in disease phenotype and insufficient consideration of lesion location and the coexistence of endometriosis. Given that endometriosis is a well-recognized confounder in reproductive outcome research, as it can independently impair implantation and fertility, we treated the presence of ovarian endometrioma and pelvic endometriosis as key confounding factors in the present study and incorporated them into multivariate models when evaluating ART indication and live birth outcomes within each adenomyosis subtype. Taken together, our findings suggest that intrinsic and extrinsic adenomyosis represent distinct clinical phenotypes with different reproductive profiles, and that differences in lesion location and endometriosis status may partly account for the subtype-specific patterns observed in ART utilization and live birth outcomes.

Conversely, in the indeterminate subtype, although the live birth rate was significantly lower and the adenomyosis lesion thickness was the greatest among the subtypes, lesion thickness was not significantly associated with ART or live birth outcomes. This may reflect structural features—such as lesion diffuseness and proximity to the endometrium—that complicate treatment and worsen the reproductive prognosis.

In the extrinsic subtype, lesion thickness was significantly greater in both patients with and without prior ART, and among those who did not achieve live birth compared with those who did. Our multivariate analyses also identified lesion thickness as a significant predictor of live birth outcomes, suggesting that with the extrinsic subtype, lesion volume has a direct impact on reproductive outcomes. This finding suggests that even slight increments in lesion size may have a measurable impact on live birth outcomes, emphasizing the clinical importance of lesion burden quantification in patients with adenomyosis. This finding implies that with the extrinsic subtype, using the appropriate intervention targeting lesion volume could improve live birth rates. In this subtype, lesions are located away from the endometrial cavity and often extend toward the posterior uterine wall [8]. This anatomical feature not only allows for the selective resection of adenomyosis but also provides an opportunity for the simultaneous resection of endometriotic lesions, which frequently occur in the same posterior region.

From a pathophysiological perspective, adenomyosis has been proposed to impair implantation through altered uterine peristalsis, chronic inflammation, and reduced endometrial receptivity [20], while concomitant endometriosis may further exacerbate implantation failure via inflammatory cytokines, progesterone resistance, and impaired embryo–endometrium crosstalk [21]. Therefore, the coexistence of endometriosis may modify the impact of adenomyosis on reproductive outcomes, particularly in the extrinsic subtype where both conditions frequently overlap. Importantly, to minimize this potential confounding effect, ovarian endometrioma and pelvic endometriosis were included as covariates in all multivariate analyses, allowing the subtype-specific associations observed in this study to be interpreted independently of coexisting endometriosis. In particular, DE has been reported in multiple studies to increase the risk of obstetric complications such as placenta previa and postpartum hemorrhage (PPH) [22–24]. Although it remains controversial whether surgical treatment for endometriosis can reduce these risks, our study suggests that the excision of pelvic lesions in severe endometriosis may lower the risk of placental abnormalities [25], indicating the need for further investigation.

Recently, since there have also been reports suggesting that adenomyomectomy may improve the pregnancy and miscarriage rates [26] and reduce the risk of obstetric complications [27–29], given that lesions of the extrinsic type are often localized and situated away from the endometrial cavity, surgical intervention—including concurrent endometriotic lesion resection—may be a feasible and effective option in selected cases, and it warrants further investigation by MRI-based subtype.

For the indeterminate subtype, our multivariate analysis identified the presence of ovarian endometrioma as a factor independently associated with reduced live birth rates. The presence of endometrioma may reflect advanced pelvic endometriosis, and it is often linked to diminished ovarian reserve due to its negative impact on ovarian function, including reduced AMH levels [30]. Therefore, in addition to the impairment in endometrial receptivity caused by adenomyosis, poor embryo quality resulting from ovarian dysfunction may contribute to lower live birth rates with this subtype.

As for the intrinsic subtype, women who achieved live birth were, paradoxically, older than those who did not. This counterintuitive result may be explained by the hypothesis that intrinsic adenomyosis is more prevalent among multiparous women [12]. It has been suggested that the disruption of the endometrial–myometrial interface, due to prior deliveries or procedures such as dilation and curettage, facilitates the development of intrinsic adenomyosis [31, 32]. While this condition may impair fertility once fully developed, it does not necessarily preclude the possibility of achieving a live birth over the course of a woman’s reproductive lifespan. These findings also underscore the importance of avoiding blind or aggressive intrauterine procedures that may damage the endometrium, potentially preventing the onset of intrinsic adenomyosis.

Although age is a well-known determinant of fertility [33], its effect varied across adenomyosis subtypes in the present study. Importantly, age was included as an adjusting variable in all multivariate analyses, and therefore the observed subtype-specific associations with ART utilization and live birth should be interpreted as independent of age-related effects.

Nevertheless, this study has several limitations that should be acknowledged. First, in many cases, ART treatment, pregnancy, and delivery preceded surgery. As a result, the state of adenomyosis evaluated by MRI may not accurately reflect the pathological condition during pregnancy. In addition, the interval between MRI examination and the last pregnancy was relatively long in many cases, which may limit the validity of using adenomyotic lesion thickness as a direct indicator of pregnancy outcomes. At the very least, it is not appropriate to evaluate the prognosis of pregnancy based on absolute thickness values. Therefore, in this study, we assessed the impact of adenomyosis by evaluating how each 1 mm increase in lesion thickness was associated with pregnancy outcomes. Therefore, the findings of this study may not be directly applicable to pregnancy outcomes, and they should be interpreted cautiously.

Second, the retrospective design of this study and the relatively small number of patients within each MRI-defined subtype limit the statistical power of the analyses and may have reduced our ability to detect modest associations. In addition, due to the cross-sectional nature of this study, we were unable to evaluate the natural history of adenomyosis or potential longitudinal transitions among MRI-defined subtypes. Whether and how adenomyosis subtypes change over time remains an important unanswered question and should be addressed in future prospective, longitudinal studies. Although multivariate analyses were performed, residual confounding cannot be fully excluded, and the results should be interpreted as exploratory rather than definitive. Third, since this study included only surgical cases, most patients had relatively severe disease. This may not be representative of the general population of patients with adenomyosis, and selection bias cannot be ruled out. Considering these limitations, prospective cohort studies targeting patients who undergo MRI before infertility treatment or pregnancy should be conducted in the future to produce more robust findings that can lead to more solid conclusions.

## Supplementary Information

Below is the link to the electronic supplementary material.

Supplementary file1 (XLSX 10 KB)

## Data Availability

No datasets were generated or analysed during the current study.

## References

[1] Galati G, Ruggiero G, Grobberio A, Capri O, Pietrangeli D, Recine N, Vignali M, Muzii L (2024) The role of different medical therapies in the management of adenomyosis: a systematic review and meta-analysis. J Clin Med 13(11):3302. 10.3390/jcm13113302 38893013 PMC11172524

[2] Selntigia A, Molinaro P, Tartaglia S, Pellicer A, Galliano D, Cozzolino M (2024) Adenomyosis: an update concerning diagnosis, treatment, and fertility. J Clin Med 13(17):5224. 10.3390/jcm13175224 39274438 PMC11396652

[3] Szubert M, Koziróg E, Olszak O, Krygier-Kurz K, Kazmierczak J, Wilczynski J (2021) Adenomyosis and infertility: review of medical and surgical approaches. Int J Environ Res Public Health 18:1235. 10.3390/ijerph18031235 33573117 PMC7908401

[4] Pados G, Gordts S, Sorrentino F, Nisolle M, Nappi L, Daniilidis A (2023) Adenomyosis and infertility: a literature review. Medicina (Kaunas) 59(9):1551. 10.3390/medicina59091551 37763670 PMC10534714

[5] Cozzolino M, Tartaglia S, Pellegrini L, Troiano G, Rizzo G, Petraglia F (2022) The effect of uterine adenomyosis on ivf outcomes: a systematic review and meta-analysis. Reprod Sci 29:3177–3193. 10.1007/s43032-021-00818-6 34981458

[6] Mandelbaum RS, Melville SJF, Violette CJ, Guner JZ, Doody KA, Matsuzaki S et al (2023) The association between uterine adenomyosis and adverse obstetric outcomes: a propensity score-matched analysis. Acta Obstet Gynecol Scand 102:833–842. 10.1111/aogs.14581 37087741 PMC10333655

[7] Tsikouras P, Kritsotaki N, Nikolettos K, Kotanidou S, Oikonomou E, Bothou A et al (2024) The impact of adenomyosis on pregnancy. Biomedicines 22(12):1925. 10.3390/biomedicines12081925 PMC11351718 39200389

[8] Chen LS, Chen X, Song JR, Zhuang YL, Xie X, Liu SH et al (2024) Impact of adenomyosis on pregnancy outcomes: a retrospective consecutive cohort study. Eur Rev Med Pharmacol Sci 28:577–583. 10.26355/eurrev_202401_35055 38305602

[9] Moawad G, Fruscalzo A, Youssef Y, Kheil M, Tawil T, Nehme J et al (2023) Adenomyosis: an updated review on diagnosis and classification. J Clin Med 21(12):4828. 10.3390/jcm12144828 PMC10381628 37510943

[10] Ottolina J, Villanacci R, D’Alessandro S, He X, Grisafi G, Ferrari SM et al (2024) Endometriosis and adenomyosis: modern concepts of their clinical outcomes, treatment, and management. J Clin Med 9(13):3996. 10.3390/jcm13143996 PMC11277467 39064036

[11] Chapron C, Vannuccini S, Santulli P, Ambrosini G, Di Donato N, Petraglia F (2020) Diagnosing adenomyosis: an integrated clinical and imaging approach. Hum Reprod Update 26(3):392–411. 10.1093/humupd/dmz049 32097456

[12] Kishi Y, Suginami H, Kuramori R, Yabuta M, Suginami R, Taniguchi F (2012) Four subtypes of adenomyosis assessed by magnetic resonance imaging and their specification. Am J Obstet Gynecol 207(2):114.e1–7. 10.1016/j.ajog.2012.06.027 22840719

[13] Hashimoto A, Iriyama T, Sayama S, Okamura A, Kato K, Fujii T, Kubota K, Ichinose M, Sone K, Kumasawa K, Nagamatsu T, Hirota Y, Osuga Y (2023) Differences in the incidence of obstetric complications depending on the extent and location of adenomyosis lesions. J Matern Fetal Neonatal Med 36(2):2226789. 10.1080/14767058.2023.2226789 37787637

[14] Shi J, Wu Y, Li X, Gu Z, Zhang C, Yan H, Dai Y, Leng J (2023) Effects of localization of uterine adenomyosis on clinical features and pregnancy outcomes. Sci Rep 13:14714. 10.1038/s41598-023-40816-z 37679426 PMC10485030

[15] Rees CO, Nederend J, Mischi M, van Vliet HAAM, Schoot BC (2021) Objective measures of adenomyosis on MRI and their diagnostic accuracy — a systematic review & meta-analysis. Acta Obstet Gynecol Scand 100(8):1377–1391. 10.1111/aogs.14139 33682087

[16] Dueholm M, Lundorf E (2007) Transvaginal ultrasound or MRI for diagnosis of adenomyosis. Curr Opin Obstet Gynecol 19:505–512. 10.1097/GCO.0b013e3282f1bf00 18007126

[17] Jain S, Kumar K, Shukla RC, Jain M (2023) Diagnostic role of transvaginal sonography and magnetic resonance imaging in adenomyosis of the uterus and its correlation with histopathology. J Midlife Health 14:34–41. 10.4103/jmh.jmh_230_22. 37680374 PMC10482023

[18] Alson S, Henic E, Hansson SR, Sladkevicius P (2024) Correlation of adenomyosis features to live birth rates after the first IVF/ICSI treatment, when using the revised Morphological Uterus sonographic assessment group definitions. Acta Obstet Gynecol Scand 103:2540–2553. 10.1111/aogs.14986 39382305 PMC11609986

[19] Liang Y, Zhu M, Sun F, Liu X, Liu J, Zhang J (2025) Phenotypic heterogeneity in adenomyosis: internal and external subtypes. Arch Gynecol Obstet 312:1667–1678. 10.1007/s00404-025-08166-5. 40886209 PMC12589224

[20] Squatrito M, Vervier J, Bindels J, Bernet L, Blacher S, Nisolle M et al (2024) Impaired fertility in adenomyosis: a murine model reveals endometrial receptivity and progesterone resistance imbalances. Reproduction 167:e240019. 10.1530/REP-24-0019 38451875 PMC11056956

[21] Tang HC, Lin TC, Wu MH, Tsai SJ (2024) Progesterone resistance in endometriosis: a pathophysiological perspective and potential treatment alternatives. Reprod Med Biol 23:e12588. 10.1002/rmb2.12588 38854774 PMC11157498

[22] Munshi H, Barada N, Anand S, Gajbhiye RK (2025) Pregnancy outcomes in women with different endometriosis lesion types: a review of current evidence. J Obstet Gynaecol Res 51:e16321. 10.1111/jog.16321 40384629

[23] Uchida S, Fukuhara R, Yokoyama M, Akaishi A, Iino K, Yokota M, Yokoyama Y (2024) Association between endometriosis and perinatal complications: a case-control study. BMC Pregnancy Childbirth 24:537. 10.1186/s12884-024-06724-4 39143505 PMC11325749

[24] Bean EMR, Knez J, Thanatsis N, De Braud L, Taki F, Hirsch M, David A, Jurkovic D (2024) Obstetric outcomes in women with pelvic endometriosis: a prospective cohort study. Fertil Steril 122(4):696–705. 10.1016/j.fertnstert.2024.05.162 38838806

[25] Ono Y, Furumura K, Yoshino O, Ota H, Sasaki Y, Hidaka T, Fukushi Y, Hirata S, Yamada H, Wada S (2022) Influence of laparoscopic surgery for endometriosis and its recurrence on perinatal outcomes. Reprod Med Biol 21(1):e12456. 10.1002/rmb2.12456 35414762 PMC8986974

[26] Liu L, Wang T, Li Y, Tian H, Zhou C, Cai W (2025) Reproductive outcomes after fertility-sparing interventions for symptomatic adenomyosis: a systematic review and meta-analysis. BMC Pregnancy Childbirth 25(1):1178. 10.1186/s12884-025-08323-3 41206447 PMC12595728

[27] Ono Y, Ota H, Fukushi Y, Tagaya H, Okuda Y, Yoshino O, Yamada H, Hirata S, Wada S (2023) Effectiveness of laparoscopic adenomyomectomy on perinatal outcomes. Gynecol Minim Invasive Ther 12(4):211–217. 10.4103/gmit.gmit_45_22 38034106 PMC10683966

[28] Kwack JY, Lee SJ, Kwon YS (2021) Pregnancy and delivery outcomes in the women who have received adenomyomectomy: performed by a single surgeon by a uniform surgical technique. Taiwan J Obstet Gynecol 60:99–102. 10.1016/j.tjog.2020.11.015 33495018

[29] Sayama S, Iriyama T, Hashimoto A, Suzuki K, Ariyoshi Y, Yano E, Toshimitsu M, Ichinose M, Seyama T, Sone K, Kumasawa K, Hirota Y, Osuga Y (2023) Possible risks and benefits of adenomyomectomy on pregnancy outcomes: a retrospective analysis. AJOG Glob Rep 3(4):100265. 10.1016/j.xagr.2023.100265 37771974 PMC10523262

[30] Yılmaz Hanege B, Güler Çekıç S (2019) Ata B (2019) Endometrioma and ovarian reserve: effects of endometriomata per se and its surgical treatment on the ovarian reserve. Facts Views Vis Obgyn 11(2):151–157 31824636 PMC6897522

[31] Khan KN, Fujishita A, Mori T (2022) Pathogenesis of human adenomyosis: current understanding and its association with infertility. J Clin Med 11(14):4057. 10.3390/jcm11144057 35887822 PMC9316454

[32] Rossi M, Vannuccini S, Capezzuoli T, Fambrini M, Vannuzzi V, Donati C (2022) Mechanisms and pathogenesis of adenomyosis. Curr Obstet Gynecol Rep 11:95–102. 10.1007/s13669-022-00326-7

[33] Delbaere I, Verbiest S, Tydén T (2020) Knowledge about the impact of age on fertility: a brief review. Ups J Med Sci 125(2):167–174. 10.1080/03009734.2019.1707913 31964217 PMC7721003

